# The Impact of the Wavelength and Its Transmittance on the Visual Evoked Potentials, at Baseline, and under the Effect of Six Monochromatic Filters Used for Visual Treatments

**DOI:** 10.3390/s23115227

**Published:** 2023-05-31

**Authors:** Danjela Ibrahimi, Enoé Crúz-Martínez, Guillermo Valencia Luna, Josué Romero Turrubiates, Juvenal Rodríguez-Reséndiz

**Affiliations:** 1 Facultad de Medicina, Universidad Autónoma de Querétaro, Santiago de Querétaro 76176, Mexico; 2 Facultad de Ingeniería, Universidad Autónoma de Querétaro, Santiago de Querétaro 76010, Mexico; 3 Hospital Infantil Teletón de Oncología, Anillo Vial Junipero Serra 1999, Santiago de Querétaro 76140, Mexico; enoecruzmtz@gmail.com; 4 Metrólogo del Laboratorio de Propriedades Ópticas de los Materiales de la Dirección Óptica y Radiometría, del Centro Nacional de Metrología, El Marqués 76246, Mexico; gvalenci@cenam.mx; 5 Facultad de Psicología, Universidad Autónoma de Querétaro, Santiago de Querétaro 76010, Mexico; j.romeroturrubiates@gmail.com

**Keywords:** visual-evoked potentials, neural activity, monochromatic filter, wavelength, transmittance

## Abstract

Purpose: This is an observational, non-invasive study which measures the VEPs of twelve individuals, at baseline, and under the effect of six monochromatic filters used in visual therapy, to understand their effect on neural activity to propose successful treatments. Methods: Monochromatic filters were chosen to represent the visible light spectrum, going from red to violet color, 440.5–731 nm, and light transmittance from 19 to 89.17%. Two of the participants presented accommodative esotropia. The impact of each filter, differences, and similarities among them, were analyzed using non-parametric statistics. Results: There was an increase on the N75 and P100 latency of both eyes and a decrease was on the VEP amplitude. The neurasthenic (violet), omega (blue), and mu (green) filter had the biggest effects on the neural activity. Changes may primarily be attributable to transmittance (%) for blue-violet colors, wavelength (nm) for yellow-red colors, and a combination of both for the green color. No significant VEPs differences were seen in accommodative strabismic patients, which reflects the good integrity and functionality of their visual pathway. Conclusions: Monochromatic filters, influenced the axonal activation and the number of fibers that get connected after stimulating the visual pathway, as well as the time needed for the stimulus to reach the visual cortex and thalamus. Consequently, modulations to the neural activity could be due to the visual and non-visual pathway. Considering the different types of strabismus and amblyopia, and their cortical-visual adaptations, the effect of these wavelengths should be explored in other categories of visual dysfunctions, to understand the neurophysiology underlying the changes on neural activity.

## 1. Introduction

Monochromatic filters have been used to treat light sensitivity in patients with traumatic brain injuries [1], change the visual-evoked potentials (VEPs) in children diagnosed with visual stress [2], improve perception of patients with symptoms of visual processing disorder [3], and reduce cortical hyper-activation in patients who suffer from migraine [4], suggesting that the neural activity of these patients, may be altered by wavelength-dependent processes. Taking into consideration the cortical origin of these conditions and the possibility to modulate the brain activity using different wavelengths, the same principle could be applied for patients with strabismus and amblyopia (SA), where abnormalities in first (luminance-based) and second-order (texture-based) processing of visual information [5], as well as the gray and white matter of the brain [6,7,8], have been reported. Strabismus is a visual disorder that affects 2–5% of preschool and school-aged children [9], whereas amblyopia affects 1–4% of the population [10]. Deficit in ocular-motricity [11] and binocularity [12], are the most common findings in SA patients. In the present, phototherapy, which employs a combination of two or more monochromatic filters, has been empirically used as part of the visual therapy treatment of SA patients [13,14], but it is the first time that VEPs are used to analyze the effect of these filters, with the goal of understanding their potential to change the neural activity and propose successful treatments to patients with visual dysfunctions. The evoked potentials are bioelectric signals produced by the central nervous system (CNS) and triggered by an external event [15,16]. VEPs are useful in assessing the functional integrity of the visual pathway in adults and children [17]. An undamaged visual pathway is followed by normal VEPs, whereas dysfunctions anywhere in the visual system can trigger abnormal VEPs. In patients with SA, abnormal VEPs not only in the amblyopic/strabismic eye, but in the fellow eye as well has been reported [18,19]. Problems lie in motion processing [20], color onset [21], interocular suppression [22], deficits in visual function across the visual field [23] and reduced retinal activity [24], among others. Therefore, using science, to find treatments for these conditions, should be a concern of visual health professionals. This study recorded, analyzed, and compared the VEPs of twelve participants, at baseline, and under the effect of six different monochromatic filters designed to treat SA patients. Filters were chosen to represent the visible light spectrum, going from red to violet color, 440.5–731 nm, and light transmittance from 19–89.17%. To determine, if the recorded data were only specific and unique to visually-normal individuals, two patients with accommodative esotropia were included in this analysis. This type of strabismus is considered to present less cortical-visual adaptations than others. Research on spectral filters designed to treat light-sensitivity, has reported that the effect of gray and yellow color on VEPs is primarily attributable to luminance, whereas blue and red ones to specific spectral effects [25]. Likewise, changes in the amplitude of visual evoked potential, in children with visual stress and symptoms of headache, have been recorded under the effect of colored lenses [2]. There are no VEP studies in the literature that report the effect on the neural activity of monochromatic filters designed to treat SA patients, to propose posteriorly successful treatments based on scientific evidence. What we know from quantitative electroencephalography studies, however, is that light stimulation and phototherapy which employs combination of two or more of these wavelengths can modulate the Alpha-wave, interhemispheric connections, anteroposterior gradient, and brain coherence [26,27], and functional connectivity patterns on brain networks measured with fMRI, are light-dependent [28]. Therefore, we expect to see the effect of these monochromatic filters on the visual pathway, which connects the eye to the cortex and modulates neural activity. In our study, six different monochromatic filters, simulating the visible light spectrum, represented by their wavelength and transmittance were used: Neurasthenic (440.5 nm/36.02%), Omega (446.5 nm/19%), Depressant (445 nm/79.5%), Mu (526.5 nm/34.76%), Stimulant (592 nm/89.17%), and Alpha (731 nm/81.80%). Nomenclature of the filters was proposed by Dr. Spitler [29]. Seven VEPs were recorded for each participant. Data were compared through the statistical analysis to understand the neurophysiology underlying the neural activation of participants and help create a therapy based on evidence.

## 2. Methods

This is an observational non-invasive study which analyzes and compares the VEPs of twelve participants at baseline and under the effect of six different monochromatic filters. Filters were chosen to represent the visible light spectrum, going from red to violet color, 440.5–731 nm, and light transmittance from 19 to 89.17%. Combinations of these wavelengths have been empirically used by visual health professionals to treat patients with strabismus, amblyopia, and other visual dysfunctions [13,14]. The goal of this study was to understand the impact of a specific wavelength on the neural activity of the participants, and shed light on the implication of its use on the human brain. Filters’ characterization was performed at The National Center of Metrology (NCM). VEPs were recorded at the Santo Tomás Hospital by the neurophysiologist in charge, and the neuro-optometric evaluation took place at Brain Vision & Learning Center, Querétaro, México. The study conformed to the principles of the Declaration of Helsinki. Consent from the participants and parents was obtained before any procedure was performed.

**Figure 1:**
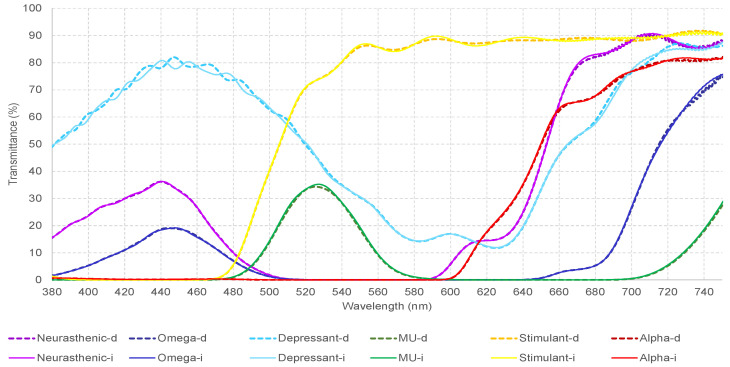
Represents the measured spectral transmittance (from 380 to 750 nm) of the monochromatic filters used in this research. The colors on the graphs, represent the colors of each filter. Additionally, r refers to the right lens and l to the left one.

(i) Medical history and visual efficacy analysis:
  Ten visually-normal individuals and two patients with accommodative esotropia participated in this study. Visually-normal participants are essential to understand how a chosen variable can modify their neural activity, to posteriorly apply it to other categories of individuals, such as SA patients. Additionally, patients with accommodative esotropia were chosen for this analysis, as it is considered to present fewer cortical-visual adaptations when compared to other types of strabismus.
The process of evaluation followed four steps:
  Step one: detailed personal medical history of the patient and their family background.
  Step two: determine the best optical prescription under the cycloplegic effect of 1% tropicamide [22] and subjective refraction afterwards.
  Step three: Evaluate the visual efficacy using the following motor and sensorial tests:
  (1) Visual acuity at 40 cm and 3 m using Bailey–Lovie charts (logMar).
  (2) Stereopsis at 40 cm using the Random-Dot 2 test which goes from 500 (gross) to 12.5 (fine) seconds of arc.
  (3) Cover–Uncover test at near (40 cm) and far fixation (6 m), to determine the magnitude and direction of strabismus, using a translucent occluder and prism bar.
  (4) Alternate Cover Test using a translucent occluder and prism bar for the magnitude and direction of phorias: at near fixation (40 cm) using a 20/30 single letter on the Gulden fixation stick, and at distance (3 m), by isolating a 20/30 letter on the distance visual acuity chart.
  (5) Worth dot test at 33 cm and 3 m respectively to evaluate flat fusion and suppression.
  (6) Monocular estimated method retinoscopy at 40 cm (MEM) as a complementary test to determine the best optical prescription.
  Step four: VEPs at baseline and under the effect of the six monochromatic filters were recorded at Santo Tomas Hospital by Dr. Enoé Cruz-Martínez, specialist in neurophysiology.
  These data were necessary to understand the functionality of the visual system and its best performance. The monochromatic filters used in this study correspond to the following colors: Alpha = red, Stimulant = yellow, Mu = green, Depressant = light blue, Omega = dark blue, and Neurasthenic = violet. Filters’ characterization (wavelength and transmittance) was performed at the National Center of Metrology. All tests were performed by the same examiner, Dr. Danjela Ibrahimi, specialist in vision and child development. The above-mentioned clinical evaluation can be found in the Appendix A. Additionally, detailed information about the visual efficacy exam can be found in [30].
(ii) VEPs recordings:
  The 0.9 version of Viking software was used to record the pattern-reversal VEPs:
  (i) Patients seated at 1 m from a 17″ monitor;
  (ii) Contrast between black and white squares of 90%, 20${}^{\prime }$ check size, and luminance of 80 $\frac{cd}{m^{2}}$;
  (iii) Stimulation frequency of 2.1 Hz;
  (iv) Band filter of 1–250 Hz (low - and - high pass filter; is predetermined and used to avoid artefacts);
  (v) Window analysis of 500 milliseconds (ms);
  (vi) Data was averaged 250 times;
  (vii) Patient’s fixation was monitored by the doctor in charge and data were collected only under fixation.
  A pattern-reversal-elicited VEP waveform comprises a negative peak, N75, which occurs at about 75 ms (records the time needed for the visual stimulus to reach the thalamus), followed by a positive peak, P100, which occurs at about 100 ms (time the visual stimulus reaches the visual cortex, Brodmann area 17), and a second negative peak, N145, that occurs at 145 ms (time the visual stimulus reaches the association areas of the visual cortex, Brodmann areas 18 and 19). For this research, the VEPs were analyzed considering the P100 latency (measured from 0 ms to the highest point of the peak), the N75 latency (measured from 0 ms to the highest point of peak), the peak-to-peak or VEP amplitude (PP Amplitude 75–100) measured from peak N75 to P100 and is expressed in microvolts (μV), and the LAT DIFF LT-RT (difference between the P100 latency of the right and left eye expressed in ms). VEPs were recorded monocularly, the P100 latency being the parameter of most interest, as it represents the reaching time of the visual stimulus at the V1. Research on VEPs of the strabismic/amblyopic eye compared to the sound eye can be found in the literature, but no previous studies report how these specific wavelengths, designed to treat visual dysfunctions, can modulate the neural activity of the individuals.
(iii) Filters’ characterization:
  The spectral transmittance of each monochromatic filter (for both, the right and left lens) was characterized using a double-beam UV-VIS-NIR spectrophotometer, VARIAN brand, Cary 5000 model, owned by the National Center of Metrology, with high precision and accuracy. It is calibrated to the magnitudes of transmittance expressed in percentage (%) and wavelengths expressed in nanometers (nm). Both measurements are traceable to the National Spectral Transmittance, Absorbance and Reflectance Standard maintained by the NCM [31]. The calibration uncertainty for transmittance and wavelength measurements of this spectrophotometer is shown in Table 1:

The characterization of each filter was performed using an incident beam geometry of 90 degrees to the central useful area of the lens surface, taking air as a reference. The dimension of the incident beam is approximately 1 mm × 10 mm, directed towards the outer surface of the lens. The parameters used for the characterization of the monochromatic filters, are illustrated in Table 2. Additionally, refer to Figure 1 for the spectral transmittance of each filter.

**Figure 2:**
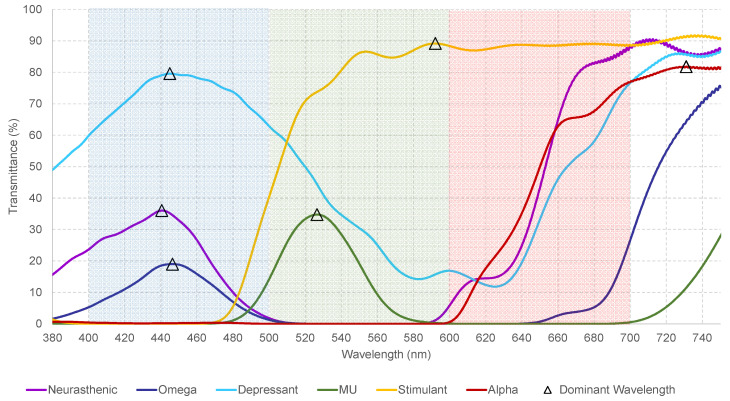
Represents the measured spectral transmittance (from 380 to 750 nm) of the dominant wavelength, represented by the triangle. The colors on the graphs, represent the colors of each filter. The dominant wavelength, identified for each measured filter as “the wavelength with the maximum transmittance in the measured spectra”, falls within the ranges given in the Visible Light Spectrum Color (Table 4) and matches the color we perceive in the filter.

These measurements allow knowing and establishing the transmittance value expressed as a percentage for each measured wavelength from 380.0 nm to 750.0 nm with increments of 0.5 nm. Table 3 shows the wavelength of each monochromatic filter used in the study. Figure 2 displays the measured spectral transmittance at certain ranges.

## 3. Results

Ten visually-normal participants and two patients with accommodative esotropia were included in this study.

Patients with accommodative esotropia: two sisters with accommodative esotropia (the deviation is completely compensated using the prescription), aged 14 and 16 years, low amblyopia of the right eye at distance and near (AV = 0.3 logMAR) and moderate stereopsis (63″ and 50″ respectively), moderate hyperopia (+3.75 and +4.00 dioptrias respectively), and astigmatism (+3.25 and +3.50 dioptrias respectively), with a familiar history of esotropia (father and uncle with the same visual dysfunction). Intermittent suppression at distance was present in both patients. Both sisters had left-eye ocular dominance.

Visually-normal participants: five male and five female participants, mean age 22.54 ± 2.51. Stereopsis value (μ = 26.4 ± 6.5) seconds of arc, with flat fusion at distance and near. All participants presented exophoria at near (μ = 9 ± 2.87 prismatic dpts). From the total, two of them were myopic (μ = −0.75 ± 0.35 dpt), the other two had hyperopia (μ = 0.87 ± 0.52 dpt), and one participant presented pure astigmatism (−1.25 dpt). The other five did not have any refractive error. Right-eye ocular dominance was found in nine of them and of the left eye only in one.

The parameters of interest in this research were the N75 and P100 latencies expressed in milliseconds (ms), the peak-to-peak or VEP amplitude (PP Amp 75–100) expressed in microvolts (μV), and the difference between the P100 latency value of the left and right eye (LAT DIFF LT-RT) expressed in milliseconds (ms). In the field of neurophysiology, the latency value represents the velocity of the transmission (wiring) of the visual stimulus. It signifies that the generation of an impulse can happen in a shorter or longer time, resulting in a slower or faster visual pathway. Transmission refers to the myelinated state of the axons. The higher the peak value, the slower the conduction is and vice versa. Demyelinated visual conditions are followed by high latencies. The peak-to-peak amplitude represents the number of axons which are being activated by the visual stimulus. A higher amplitude means more activated axons and more synaptic connections. Axons refer to the number of fibers that are activated and fire together. Axonal compromised visual conditions are followed by slower peak-to-peak amplitude. For both parameters (latency and amplitude) a change of up to 30% from its baseline value is considered neurologically important. The interocular measurements represent the difference between the P100 latency of the left and right eye. Differences ≥ 2 ms between eyes are considered the norm. Higher differences signify that an eye is conducting faster/slower than the other one.

The analysis of our results was divided into two phases:

Phase One:

To determine if the gathered data were only specific and unique to visually-normal individuals, the VEP recordings of two visually-normal participants were compared to those of two strabismic patients. Accommodative esotropia was chosen for comparison as it presents less cortical-visual adaptations when compared to other types of strabismus. To make the comparison valid, variables such as gender, age, socioeconomic status, and educational level were considered.

In this phase, VEPs were analyzed and compared according to their neurophysiological significance, emphasizing their medical aspect. Table 5 and Table 6 present the monochromatic filters that cause changes greater than 30% compared to the baseline value, for the strabismic and non-strabismic participants.

What stands out from Table 5a,b is the significant decrease of the VEP amplitude under the effect of most filters. These results indicate that monochromatic filters impact the axonal activation, by diminishing the number of connected fibers. Another significant result is the impact of monochromatic filters on the interocular measurements (LAT DIFF LT-RT), where values >2 ms are considered abnormal. Interestingly, while an important increment in the LAT DIFF LT-RT values is observed for the 1st patients (the conduction of the visual stimulus occurs at different velocities), a decrease is recorded for the 2nd one (both eyes are transmitting at similar velocities).

Additionally, a weaker impact was observed for the N75 and P100 latency, its effect being considerable only for the N75 latency of the 1st patient. The differences found between these patients are consistent with what is known at neurological level: every strabismic brain is unique, as are their cortical adaptations.

Similar VEPs were obtained for the non-strabismic patients. Table 6a,b, shows a significant decrease of the VEP amplitude and an important increment in the LAT DIFF LT-RT values under the effect of most filters. Likewise, a very weak impact was observed on the N75 and P100 latency, being neurologically significant only for the N75 latency of the 1st participant.

From Table 5 and Table 6 it can be concluded that, when the visual system is exposed to monochromatic wavelengths, the greatest response is obtained under the effect of green, violet, and blue colors. Additionally, the visual pathway of patients with strabismus reacts to more filters than that of non-strabismic participants, which could be explained by the fact that a strabismic brain is always looking for a stimulus to react to and improve its functionality. Moreover, changes on the N75 latency (the time needed for the visual stimulus to reach the thalamus) suggest that the non-visual pathway is the most affected by the exposure to monochromatic wavelengths. Finally, the similarities found among participants, suggest that patients with accommodative esotropia compensate to some extent for their sensorial deficits provoked by the strabismus.

Phase Two:

Statistical analysis: To detect statistically significant differences between the analyzed variables, non-parametric tests were performed using the SPSS Statistics Base 25.0 program. The confidence level (CI) used in this study was 95%, with alpha = 0.05 (α = 0.05). The Friedman test was used as an alternative to the repeated measures ANOVA. The Wilcoxon signed-rank test with Bonferroni adjustment for multiple comparison was performed to find out where exactly the differences lie. Considering the similarities found on the VEPs of patients with strabismus, their data were included for the statistical analysis in this phase. At first, to determine the impact of monochromatic filters on the parameters of the VEP, the Friedman test was used as an alternative to the repeated measures ANOVA. Results are represented in Table 7.

No statistically significant differences were found for the interocular measurements (LAT DIFF RT-LT), nor the N145 latency of the right and left eye, as a dependent variable of the monochromatic filter used. Mean values and standard deviations of the above-mentioned parameters which presented statistically significant differences under the effect of monochromatic filters are shown in Table 8a,b.

**Figure 3:**
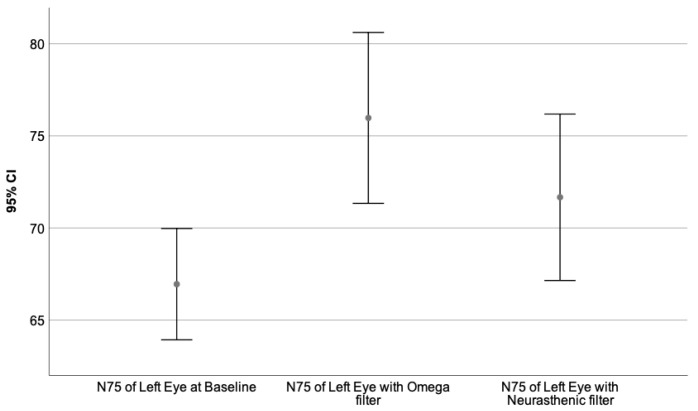
Illustrates the mean value of the N75 latency of the left eye for 95% CI, at baseline, and under the effect of the Omega and Neurasthenic filter. The X-axis shows the monochromatic filters that had a statistically significant impact on the baseline value, while the Y-axis presents the obtained values expressed in milliseconds. As can be seen by the graphics, the Omega filter had the biggest effect on the N75 latency of the left eye, by significantly incrementing its value.

**Figure 4:**
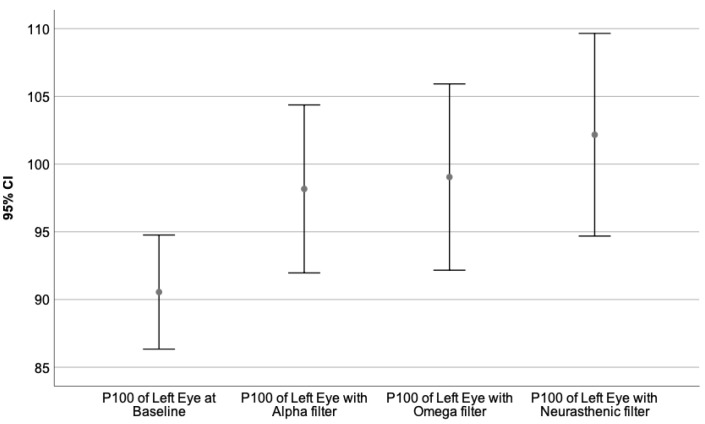
Illustrates the mean value of the P100 latency of the left eye for 95% CI, at baseline, and under the effect of the Alpha, Omega, and Neurasthenic filter. The X-axis shows the monochromatic filters that had a statistically significant impact on the baseline value, while the Y-axis presents the obtained values expressed in milliseconds. As it can be seen by the graphics, the Neurasthenic filter incremented significantly the N75 latency of the left eye.

**Figure 5:**
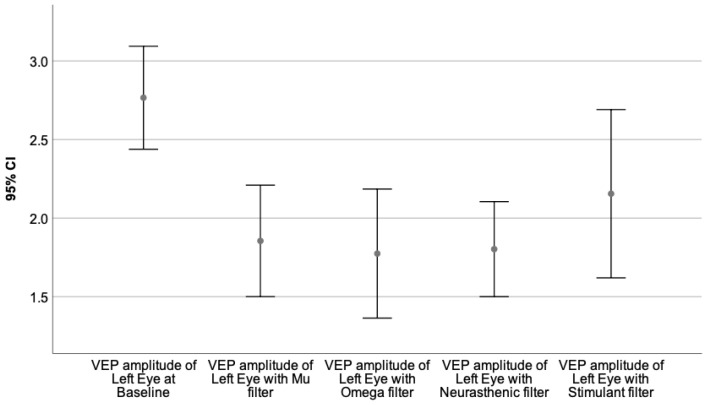
Illustrates the mean value of the VEP amplitude of the left eye for 95% CI, at baseline, and under the effect of the Mu, Omega, Neurasthenic, and Stimulant filters. The X-axis shows the monochromatic filters that had a statistically significant impact on the baseline value, while the Y-axis presents the obtained values expressed in microvolts. As can be seen from the graphics, the effect of Omega and Neurasthenic filters is almost identical. They significantly diminished the VEP amplitude when compared to its baseline value.

**Figure 6:**
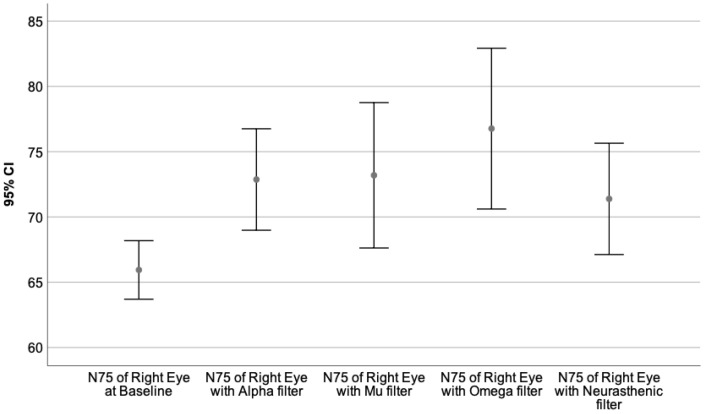
Illustrates the mean value of the N75 latency of the right eye for 95% CI, at baseline, and under the effect of the Alpha, Mu, Omega, and Neurasthenic filter. The X-axis presents the monochromatic filters that had a statistically significant impact on the baseline value, while the Y-axis shows the obtained values expressed in milliseconds. As can be seen from the graphics, the Omega filter had the biggest effect on the N75 latency value of the right eye, by causing an important increment in its value.

**Figure 7:**
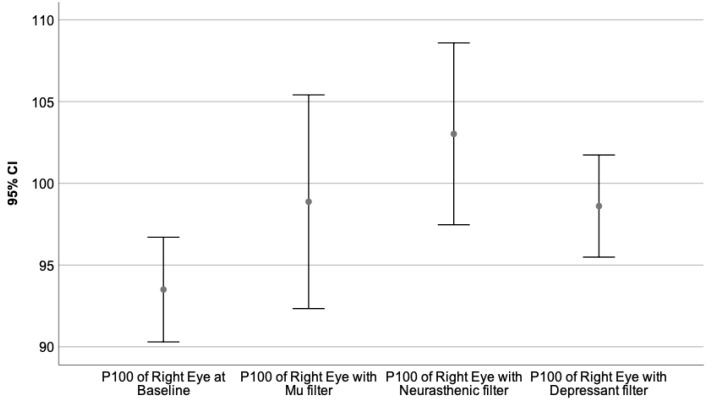
Illustrates the mean value of the P100 latency of the right eye for 95% CI, at baseline, and under the effect of the Mu, Neurasthenic, and Depressant filter. The X-axis presents the monochromatic filters that had a statistically significant impact on the baseline value, while the Y-axis shows the obtained values expressed in milliseconds. As can be seen from the graphics, under the effect of the Neurasthenic filter, the P100 latency of the right eye increased significantly.

**Figure 8:**
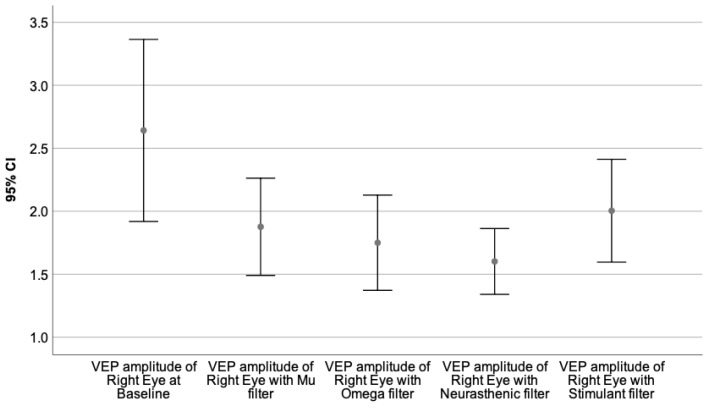
Illustrates the mean values of the VEP amplitude of the right eye for 95% CI, at baseline, and under the effect of Mu, Omega, Neurasthenic, and Stimulant filters. The X-axis shows the monochromatic filters that had a statistically significant impact on the baseline value, while the Y-axis presents the obtained values expressed in microvolts. As can be seen from the graphics, the Neurasthenic filter had the biggest effect on the VEP amplitude of the right eye, by significantly diminishing its value.

Moreover, the VEPs recorded with each monochromatic filter were compared to the baseline value, performing the Wilcoxon signed-rank test. The neural behavior under the influence of the specific wavelength is presented in Table 9. The graphical representation of the obtained results is illustrated by Figure 3, Figure 4, Figure 5, Figure 6, Figure 7 and Figure 8.

What captures our attention from the presented data is the impact of the Neurasthenic filter (violet color) on all parameters of the VEPs for both eyes. The Omega (blue color) and Mu (green color) filters provoke important changes in the neural activity of participants as well, while the rest of the filters have a weaker influence on the VEP-recorded responses.

Figure 3, Figure 4 and Figure 5 represent the VEPs parameters of the left eye that differ significantly from their baseline recording.

Figure 3 presents the N75 latency of the left eye at baseline compared to its value obtained through the Omega and Neurasthenic filter.

The P100 latency of the left eye at baseline compared to its value obtained with the Alpha, Omega and Neurasthenic filter is presented by Figure 4.

Figure 5 illustrates the VEP amplitude of the left eye at baseline compared to its value obtained with Mu, Omega, Neurasthenic, and Stimulant filters.

Figure 6, Figure 7 and Figure 8 illustrate the VEPs parameters of the right eye that differ significantly from their baseline recording.

Figure 6 presents the results of the N75 latency of the right eye at baseline compared to its value obtained with Alpha, Mu, Omega, and Neurasthenic filters.

The P100 latency of the right eye at baseline compared to its value obtained through Mu, Neurasthenic, and Depressant filters, is shown in Figure 7.

Figure 8 presents the VEP amplitude of the right eye at baseline, compared to its value obtained through Mu, Omega, Neurasthenic, and Stimulant filters.

## 4. Discussion

This was an observational non-invasive study which analyzed and compared the VEPs of twelve participants at baseline and under the effect of six different monochromatic filters. Filters were chosen to represent the visible light spectrum, going from red to violet color, 440.5–731 nm, and light transmittance from 19 to 89.17%. Phototherapy, which consists of the combination of these wavelengths, has been empirically used by visual health professionals as a complementary treatment for patients with strabismus, amblyopia, and other visual conditions [13,14]. There are no VEP studies in the literature that report the effect of these monochromatic filters which were designed to treat patients with strabismus and amblyopia, on neural activity, to propose posteriorly, successful treatments based on solid scientific evidence. However, what we know from quantitative electroencephalography studies, is that light stimulation and phototherapy which employs a combination of two or more of these wavelengths (mu-alpha, omega-neurasthenic, alpha-omega etc), can modulate the alpha-wave, interhemispheric connections, anteroposterior gradient, and brain coherence [26,27]. Likewise, a pilot study on healthy participants showed that functional connectivity patterns on brain networks measured with fMRI, are light-dependent [28]. Therefore, we expect to see the effect of these monochromatic filters on the visual pathway, which connects the eyes to the cortex and modulates neural activity. Visually-normal participants are essential to understand how a chosen variable can modify their cortical response, to apply it afterwards, to other categories of individuals, such as patients with visual problems. To determine if the gathered data were only specific and unique to visually-normal individuals, two patients with accommodative esotropia were included in this analysis, as it is considered to present less cortical-visual adaptations when compared to other types of strabismus. Research on the VEPs of the amblyopic and fellow eye are reported in the literature [18,19], but this is the first time that a study has analyzed the effect of these specific monochromatic filters which have been designed and used to treat patients with visual dysfunctions. Through our research, we aimed to shed light on whether changes in the neural activity are attributable to the specific wavelength, transmittance, or a combination of both. Considering that VEPs measure the functionality and the integrity of the visual pathway, and the visual system is the vector that connects eyes to the brain, knowing how to modulate it can be a powerful tool for visual health professionals. Therefore, by analyzing the effect of each individual filter, to understand the specific potential hidden behind its wavelength and transmittance, successful treatments for patients with visual dysfunctions can be proposed.

The discussion of our results will be conducted in two phases.

In phase one, the VEPs of two patients with accommodative esotropia and two visually-normal participants, at baseline and under the effect of monochromatic filters were analyzed and compared according to their neurophysiological significance, emphasizing the medical aspect. Firstly, we analyzed the neural activation expressed by the N75, P100, VEP amplitude, and LAT DIFF LT-RT. Secondly, we looked for differences and similarities among them, considering that we did not find other electrophysiological data on the effect of monochromatic filters in patients with strabismus and amblyopia. From our analysis, a common pattern among the four participants was found: a decrease in the VEP amplitude of both eyes under the effect of the neurasthenic filter (violet). Likewise, mu (green color) was the filter with the second-highest impact on the axonal activation of the visual pathway, followed by omega (dark blue), alpha (red), depressant (light blue), and stimulant (yellow). While the neurasthenic, mu, and omega filters always cause a decrease in the VEP amplitude value, the depressant and alpha filters can increase or decrease its value. Additionally, a decrease in the VEP amplitude under the effect of monochromatic filters was recorded in 91.7% of the cases compared to its baseline value. Changes greater than 30%, which are considered neurologically important, were found on 58.3% of the recorded data. The VEP amplitude represents the activity of neuronal populations. A larger amplitude means more activated axons, which results in more connections between neurons (synapsis), which in turn defines the neural networks and cortical plasticity [32]. Neuroplasticity is the variability of the size and type of neuronal networks using long-term potentiation or depression. The process of neuroplasticity allows the remodeling of the brain. As represented by facts, in these four participants, violet-blue-green filters acted like inhibitors, causing depression of neuronal networks [33]. A global decrease in functional connectivity in most networks was found after a minute of green and blue light exposure through a fMRI study [28], which leads us to the same conclusion, namely, that these ranges of wavelengths can modify brain networks which are arranged to perform better on tasks associated with specific cognitive demands. An increment on the LAT DIFF LT-RT was observed in 58.3% of the cases, against 37.5% that presented a decrease in its value. Only 4.2% did not show any changes. From the total, 66.7% of the data showed a difference > 2 ms between the left and right eye recording, which is considered abnormal in the field of neurophysiology. Differences of >2 ms between eyes are considered the norm. Higher differences signify that an eye is conducting faster/slower than the other one. Our findings suggest that when including monochromatic filters in the treatment program of SA patients, we could modulate the way eyes transmit visual information to the V1. This signifies that the visual stimulus reaches the 17 Brodmann area at different times, which makes us hypothesize that this could affect the integration of information at cortical level. When analyzing the N75 and P100 latency, a change greater than 30% from the baseline value is considered neurologically significant. An increment was observed in 91.7% of the cases for the N75 of the right eye, against 70.8% for the left eye. A decrease in its value was found in 8.3% of the cases for the right eye, against 29.2% for the left eye. Additionally, 75% of the cases presented an increment in the P100 latency, whereas 25% showed a decrease of it. Despite the increase and decrease in the N75 and P100 latency, changes greater than 30% were only presented in few cases (20.8% for N75 and only 4.2% for P100). In the field of neurophysiology, the latency value represents the transmission speed of the visual stimulus, which means that the generation of an impulse can happen in a shorter or longer time, resulting in a slower or faster visual pathway. Transmission refers to the myelinated state of the axons. The higher the peak value, the slower the conduction is and vice versa. Under the effect of these monochromatic filters, there is a slowness on the conduction of the visual information to the occipital cortex. Filters affected mostly the N75 latency, which represents the time the information reaches the thalamus, considered a subcortical area. Minor changes were provoked to the P100 latency, being representative of the permeability of the striated visual cortex. What we can deduct from these results is that the way to modulate the neural activity when using the visual system as a vector is by activating the visual (striate cortex) and non-visual pathway as well, which reaches the subcortical areas of the brain. Our results are in concordance with what we know about the stimulation of the brain through the monochromatic light, where human exposure to light has been demonstrated to impact both visual and non-visual components, and the use of different wavelengths affects the retinal functions, circadian rhythms, metabolic processes, sleep, mood, and growth [34,35].

In phase two, to determine the impact of monochromatic filters on the VEPs parameters of all twelve participants, the Friedman and the Wilcoxon signed-rank tests were performed.

The main difficulty we found in comparing our data to previous studies is the lack of research in this topic. It is the first time that the effect of these monochromatic filters, which have been widely used as a complementary treatment for patients with visual dysfunctions such as strabismus, amblyopia, convergence-accommodative problems, etc., on the VEPs of participants, has been analyzed. However, we found similar studies that allowed us to compare our data and draw logical conclusions. In previous research in visually-normal participants, three monochromatic filters (red, yellow, and blue) with similar wavelengths but different transmission to ours, were used [25]. The main difference was that the lens transmission in their research was a constant (each lens had a mean transmission of 39%), while in our, a variable (from 19 to 89.17%). Nevertheless, our results are in concordance with theirs, as in both studies, an increase in the N75 and P100 latencies was recorded when filters were in place (the obtained values were higher than baseline). In addition, variabilities were reported on the VEP amplitude with the addition of spectral filters, stronger under the blue filter condition. We reported similar results under the effect of omega, neurasthenic, and mu filter, (violet-blue-green color), compared to the baseline value. Based on the neurophysiology of the visual system, when the neural activity becomes more synchronized or desynchronized, an increase or decrease in the VEP amplitude is normally expected. What we know from previous research in TBI patients is that the modification of incident light may increase synchronization between neurons [1], the prescription of colored lenses in children with visual stress, increases the VEP amplitude [2], and fMRI evidence has shown reduced cortical hyper-activation in migraine patients under the effect of ophthalmic tints [4]. Likewise, in our population which was composed of visually-normal and strabismic patients, the violet-blue-green filters decreased the VEP amplitude, while the red-yellow filters had a weaker effect on the recorded values. These results can be attributable to the specific wavelength (for red-yellow colors), transmittance (blue-violet colors) and the combination of both (green color). More specifically, the omega (446.5 nm/19.00%) and depressant (445 nm/79.57%) filters differ significantly in terms of transmittance, which suggest that their activity can be transmittance-dependent. The same principle applies for the neurasthenic (440.5 nm/36.02%) and omega (446.5 nm/19.00%) filter. However, when the mu (526.5 nm/34.76 %) is compared to neurasthenic and omega filters, they differences are reflected on both, wavelength, and transmittance, which suggests that its effect can be the result of the combination of both. alpha (731 nm/81.80%) and stimulant (592/89.17%) filters on the other hand differ significantly in terms of wavelengths but show similar transmittance. Therefore, their influence on the neural activity can be mostly wavelength-dependent. As a general conclusion, we could say that the impact of blue-violet filters on the neural activity of participants is more related to their transmittance, for yellow-red colors it can be wavelength-dependent, while for the green color, it is mostly a combination of both. In another study, when ophthalmic tinted lenses (blue-purple, green-turquoise, blue-turquoise) of different transmittances (16–43%) were prescribed to patients with visual stress [2], patients with symptoms of headache and migraine presented a larger VEP amplitude when compared to the baseline value and the obtained results were mostly attributed to the transmittance than the wavelength per se. On the other hand, when the transmittance of the filter was maintained constant (39%), the effect of spectral filters was primarily attributable to luminance and in some cases, specific spectral effects [25]. When we compared our data to previous research, some points of convergence and others of divergence were found. These conclusions were expected, as in all above-mentioned studies, different wavelengths, transmittances, and samples were used. However, researchers agree that monochromatic filters can affect the neural activity and this modulation can be due to the wavelength, transmittance, or the combination of both. As researchers and clinicians, we know that the main goal of conducting research, is to understand, integrate, and apply the new information in the clinical practice. As mentioned above, the VEPs changed with filters in place. However, some parameters were more affected than others. In this present study, important neurological and significant statistical changes were found for the VEP amplitude, N75 latency of the right eye, P100 latency, and the N75 of the left eye. As known, the N75 latency presents the time needed for the visual stimulus to reach the thalamus, whereas the P100 latency shows the time the visual stimulus reaches the occipital cortex, Brodmann area 17. Therefore, the speed at which the visual stimulus reaches the striate cortex or the subcortical areas can be clinically modified by stimulating the visual and non-visual pathway, being this one, the bridge that connects the visual system to the thalamus, the integrating center of all senses, but for smell [36]. The simultaneous integration of different sensory modalities during the first years of the child’s development, is crucial for brain plasticity, and can easily be disrupted by visual dysfunctions [37]. Consequently, the use of monochromatic filters in patients with strabismus and amblyopia could be a good ally to revert what was disrupted by abnormalities of the visual system. In addition, we focused on analyzing the chromatic filters with the greatest effect on the VEPs as well. Table 9 reflects a generalized impact of the neurasthenic (violet color) followed by omega (blue color) filter, on all parameters of the VEPs. What is interesting here is the fact that both filters have a similar wavelength but differ significantly in terms of transmittance. Therefore, their effect should be transmittance-dependent. Previous research has shown the positive effects of the monochromatic blue light on cognitive performance and sleep patterns, a reason why it has been frequently used to modulate brain networks [38,39]. We strongly believe that both filters should be integrated as a complementary tool in the visual therapy program of patients with visual dysfunctions, taking into consideration that green-blue colors enhance interhemispheric connections and the brain coherence of patients with strabismus and amblyopia [26,27]. On the other hand, the green color (mu filter), proved to be another strong ally to alter the VEPs of our participants, having a comparable effect to omega and neurasthenic filters. In the field of phototherapy, the green color is considered to bring the visual system to balance [14], and research on the response to violet, blue, and green monochromatic light has reported satisfactory results at cortical and subcortical levels, after the process of exposure to these wavelengths [40]. Based on the above, that neuroimaging studies have demonstrated the positive effect of exposure to violet-blue-green monochromatic light, our VEPs confirm that visual pathways respond mostly to these range of wavelengths. Last, yellow-red colors showed a weaker effect on the VEPs of all participants. Their impact was mostly wavelength-dependent. The yellow color decreased the VEP amplitude, while the red color influenced the N75 and P100 latency. Even though they belong to a similar color spectrum, they differ significantly in their activity. Therefore, the differences could be mainly wavelength-related, and less transmittance-dependent. What we know about the combination of yellow-red colors used in visual therapy is that, even under the light stimulation, alpha-wave continued being distributed in different cortical lobes, and there was a lack of brain coherence in most patients, even after the treatment [26,27]. What could be considered a limitation to our study is the lack of a larger sample of patients with strabismus and amblyopia. The monochromatic filters we used have been especially employed to treat patients with strabismus, amblyopia, binocular dysfunctions, etc. When used in a therapy session, a combination of two or more filters is proposed to the patient, and very little research exists on their impact on the brain of strabismic and amblyopic patients. It is the first time that the effect of separate filters is measured, to understand why these specific combinations are good for some patients but not for others. In our previous studies using qEEG [26,27], we saw that phototherapy indeed modulated the brain of our patients, but with different results, especially in children and adults. Considering that strabismus is a cortical phenomenon and not just an aesthetic problem, present since childhood and affecting the quality of life of our children, understanding how monochromatic filters can be used to change their brain in a specific way so the treatment could be efficient and lasting over time should be the goal of every visual health professional. Therefore, analyzing these filters in two different samples such as strabismic and visually-normal children from 6 (the VEPs are more consistent) to 15 years old (brain networks become similar to adults), could be crucial for the future use of monochromatic filters. The biggest problem with children is their visual attention, as they easily become distracted. Considering that we used six different filters and a baseline recording, the time needed for the study is longer than they can bear. Consequently, most studies are performed in adults, and before working with children, our next step would be to repeat these measurements in strabismic adult patients and compare the data with the results we obtained in this phase. The challenge of working with patients with strabismus and amblyopia is the variability of types of strabismus, such as associated and dissociated strabismus, constant and intermittent, unilateral, alternate, each one followed by a variability of cortical-visual adaptations. Therefore, it is a necessity that more research is carried out in this field, to provide new electrophysiological data on strabismic children and adults, to compare it posteriorly to the normative population. This knowledge would open a new panorama on how to modulate the brain of these patients using different chromatic filters, to disrupt sensory and motor adaptations provoked by these visual dysfunctions.

## 5. Conclusions

This was an observational non-invasive study which analyzed and compared the VEPs of twelve participants at baseline and under the effect of six different monochromatic filters designed to treat visual dysfunctions. Filters were chosen to represent the visible light spectrum, going from red to violet color, 440.5–731 nm, and light transmittance from 19 to 89.17%. Results showed an increase in the N75 and P100 latency and a decrease in the VEP amplitude of both eyes. Therefore, modulations to the neural activity could be due to the visual and non-visual pathways. The neurasthenic (440.5 nm/36.02%), omega (446.5/19%), and mu (526.5 nm/34.76%) filters had the highest impact on the visual pathway, while stimulant, alpha, and depressant filters showed a weaker effect. Changes may be primarily attributable to the transmittance (%) for blue-violet colors, wavelength (nm) for yellow-red colors, and a combination of both for the green color. No significant VEP differences were seen in accommodative strabismic patients, which suggests that they compensate to some extent for their sensorial deficits provoked by the strabismus. Considering the different types of strabismus and amblyopia, and their cortical-visual adaptations, the effect of these wavelengths should be explored in other categories of visual dysfunctions to understand the neurophysiology underlying these modulations.

## Figures and Tables

**Table 1:** Uncertainty, *U*, for the wavelength and transmittance.

Scale	Value	*U*
Wavelength (nm)	Spectral Bandwidth 1.00 nm	0.073 nm
Transmittance (%)	0.0001–0.0100%	0.0046%
	0.1000%	0.0048%
	1.0000%	0.0066%
	10.000%	0.025%
	20.000%	0.045%
	30.000%	0.064%
	50.00%	0.10%
	70.00%	0.14%
	90.00%	0.18%

**Table 2:** Parameters used for filters’ characterization.

Parameters	Conditions
Measurement range (transmittance)	0.000% a 100.000%
Measurement range (wavelength)	380.0 nm a 750.0 nm
Scan rate	900.0 $\frac{\mathrm{nm}}{\min}$
Data interval	0.5 nm
Signal averaging time	0.033 s
Spectral bandwidth	1.0 nm

**Table 3:** Represents the wavelength of each monochromatic filter used in the study.

	Dominant Wavelength	
**Name of the Filter**	**Wavelength (nm)**	**Transmittance** **(%)**
Neurasthenic	440.5	36.02
Omega	446.5	19.00
Depressant	445	79.57
Mu	526.5	34.76
Stimulant	592	89.17
Alpha	731	81.80

**Table 4:** Shows the range of the wavelength expressed in (nm) for the Visible Light Spectrum.

Visible Light Spectrum Color		
**Color**	**Range of the Wavelength (nm)**	
Violet	380	440
Blue	440	480
Cyan	480	500
Green	500	560
Yellow	560	590
Orange	590	620
Red	620	750

**Table 5:** (a,b) Presents the monochromatic filters that cause changes greater than 30% on the parameters of the VEP compared to the baseline value for the 1st (a) and 2nd (b) patient with strabismus. Increments and decreases of the measured values greater than 30% are presented in bold.

LEFT EYE MEASUREMENTS						
	N75 LAT (ms)	B–F %	P100 LAT (ms)	B–F %	VEP amplitude 75–100 (μV)	B–F %
BASELINE	66.3		95.5		3.45	
MU	66.0	0.5−	82.0	14.2−	**1.17**	**66.1−**
NEURASTHENIC	77.3	16.5+	107.0	12.0+	**1.89**	**45.3−**
STIMULANT	**88.8**	**34.0+**	103.0	7.8+	**1.48**	**57.1−**
RIGHT EYE MEASUREMENTS						
	N75 LAT (ms)	B–F %	P100 LAT (ms)	B–F%	VEP amplitude 75–100 (μV)	B–F %
BASELINE	64.8		93.8		2.62	
MU	**86.5**	**33.5+**	106.0	13.0+	1.89	27.9−
NEURASTHENIC	77.3	19.3+	104.0	10.9+	**1.53**	**41.6−**
DEPRESSANT	69.5	7.3+	99.0	5.5+	**1.43**	**45.4−**
LAT DIFF LT-RT (ms)						
Baseline	Alpha	Mu	Omega	Neurasthenic	Stimulant	Depressant
1.75	6.50	23.50	6.00	2.75	4.50	1.00
(**a**)						
LEFT EYE MEASUREMENTS						
	N75 LAT (ms)	B–F %	P100 LAT (ms)	B–F %	VEP amplitude 75–100 (μV)	B–F %
BASELINE	68.5		81.3		2.85	
ALPHA	61.3	10.5−	85.3	4.9+	**3.87**	**35.8+**
MU	62.5	8.8-	92.0	13.2+	**1.88**	**34.1−**
OMEGA	75.8	10.7+	89.5	10.0+	**1.51**	**47.1−**
NEURASTHENIC	74.5	8.8+	89.3	9.8+	**1.22**	**57.2−**
STIMULANT	78.3	14.3+	**109.0**	**34.1+**	2.16	24.2−
RIGHT EYE MEASUREMENTS						
	N75 LAT (ms)	B–F %	P100 LAT (ms)	B–F %	VEP amplitude 75–100 (μV)	B–F %
BASELINE	60.8		89.5		3.33	
ALPHA	64.5	6.1+	87.3	2.5−	**1.88**	**43.5−**
MU	66.0	8.5+	92.0	2.8+	**1.78**	**46.5−**
NEURASTHENIC	64.0	5.3+	89.3	0.3−	**1.81**	**45.6−**
DEPRESSANT	57.5	5.4−	107.0	19.5+	**0.60**	**82.0−**
LAT DIFF LT-RT (ms)						
Baseline	Alpha	Mu	Omega	Neurasthenic	Stimulant	Depressant
8.25	2.00	0	6.25	0	1.25	8.50
(**b**)						

LAT, latency expressed in milliseconds (ms); B-F, difference between the baseline value and the one obtained using the specific filter converted to percentage (%), where the (+) sign signifies an increment in the recorded value and the (−) represents a decrease of the value; PP AMP, the amplitude measured from peak N75 to P100 expressed in microvolts (μV); LAT DIFF LT-RT, difference in the P100 latency of the left and right eye expressed in milliseconds (ms).

**Table 6:** (a,b) Shows the monochromatic filters that cause changes greater than 30% on the parameters of the VEP compared to the baseline value for the 1st (a) and 2nd (b) control participant. Increments and decreases of the measured values greater than 30% are presented in bold.

LEFT EYE MEASUREMENTS						
	N75 LAT (ms)	B–F %	P100 LAT (ms)	B–F %	VEP amplitude 75–100 (μV)	B–F %
BASELINE	57.5		92.5		2.69	
ALPHA	**76.5**	**33.0+**	106.0	14.6+	**1.52**	**43.5−**
MU	73.3	27.5+	97.3	5.2+	**1.85**	**31.2−**
OMEGA	**77.3**	**34.4+**	107.0	15.7+	**1.22**	**54.7−**
NEURASTHENIC	**75.5**	**31.3+**	101.0	9.2+	**1.74**	**35.3−**
DEPRESSANT	69.0	20.0+	90.2	2.5−	**1.81**	**32.7−**
RIGHT EYE MEASUREMENTS						
	N75 LAT (ms)	B–F %	P100 LAT (ms)	B–F %	VEP amplitude 75–100 (μV)	B–F %
BASELINE	65.8		95.0		2.27	
ALPHA	82.8	25.8+	110.0	15.8+	0.79	**65.2−**
MU	81.0	23.1+	103.0	8.4+	1.55	**31.7−**
OMEGA	78.5	19.3+	102.0	7.4+	0.89	**60.8−**
LAT DIFF LT-RT (ms)						
Baseline	Alpha	Mu	Omega	Neurasthenic	Stimulant	Depressant
2.50	4.75	5.25	5.00	6.00	3.75	9.05
(**a**)						
LEFT EYE MEASUREMENTS						
	N75 LAT (ms)	B–F %	P100 LAT (ms)	B–F %	VEP amplitude 75–100 (μV)	B-F %
BASELINE	71.3		94.8		3.18	
NEURASTHENIC	76.5	7.3+	110.0	16.0+	**2.18**	**31.5−**
DEPRESSANT	70.3	1.4−	95.3	0.5+	**4.20**	**32.0+**
RIGHT EYE MEASUREMENTS						
	N75 LAT (ms)	B–F %	P100 LAT (ms)	B–F %	VEP amplitude 75–100 (μV)	B-F %
BASELINE	69.0		97.3		3.66	
OMEGA	83.3	20.7+	110.0	13.0+	**1.77**	**51.6−**
NEURASTHENIC	79.3	14.9+	108.0	10.9+	**2.18**	**40.4−**
LAT DIFF LT-RT (ms)						
Baseline	Alpha	Mu	Omega	Neurasthenic	Stimulant	Depressant
2.50	0.25	1.00	1.75	2.50	5.25	4.75
(**b**)						

LAT, latency expressed in milliseconds (ms); B-F, difference between the baseline value and the one obtained using the specific filter converted to percentage (%), where the (+) sign signifies an increment in the recorded value and the (−) represents a decrease of the value; PP AMP, the amplitude measured from peak N75 to P100 expressed in microvolts (μV); LAT DIFF LT-RT, difference in the P100 latency of the left and right eye expressed in milliseconds (ms).

**Table 7:** Shows the results of the effect of monochromatic filters on the VEPs, obtained using the Friedman test. Statistically significant differences were found for the N75 and P100 latency and the VEP amplitude of both eyes. The $\chi^{2}$ and *p*-value depicts the strength of their impact on the neural activity of our participants.

N = 12	Left Eye		Right Eye	
	$\chi^{2}$ (6)	*p*-Value	$\chi^{2}$ (6)	*p*-Value
N75 latency (ms)	16.25	0.012	34.48	<0.001
P100 latency (ms)	15.33	0.018	13.25	0.039
VEP amplitude (μV)	26.00	<0.001	16.96	0.009

**Table 8:** (a,b) Illustrates the mean values and standard deviations of VEP parameters which presented statistically significant differences compared to their baseline value under the effect of monochromatic filters.

N = 12	Mean ± Std	Mean ± Std	Mean ± Std	Mean ± Std
	Baseline	Alpha	Mu	Omega
N75 of left eye	66.95 ± 4.76	70.07 ± 5.57	69.75 ± 7.39	75.97 ± 7.29
P100 of left eye	90.55 ± 6.63	98.17 ± 9.76	95.69 ± 9.35	99.04 ± 10.82
VEP amplitude of left eye	2.76 ± 0.52	2.53 ± 1.05	1.85 ± 0.56	1.77 ± 0.65
N75 of right eye	65.94 ± 3.52	72.87 ± 6.11	73.19 ± 8.76	76.77 ± 9.69
P100 of right eye	93.50 ± 5.04	100.12 ± 9.97	98.87 ± 10.28	100.1 ± 14.86
VEP amplitude of right eye	2.64 ± 1.14	1.94 ± 1.11	1.87 ± 0.61	1.75 ± 0.59
(**a**)				
N = 12	Mean ± Std	Mean ± Std	Mean ± Std	
	Neurasthenic	Stimulant	Depressant	
N75 of left eye	71.67 ± 7.10	71.65 ± 7.68	65.94 ± 5.38	
P100 of left eye	102.17 ± 11.78	95.29 ± 11.07	95.66 ± 5.23	
VEP amplitude of left eye	1.80 ± 0.47	2.15 ± 0.84	2.29 ± 1.32	
N75 of right eye	71.38 ± 6.70	69.37 ± 6.36	64.77 ± 5.49	
P100 of right eye	103.02 ± 8.75	97.61 ± 10.69	98.61 ± 4.91	
VEP amplitude of right eye	1.60 ± 0.41	2.0 ± 0.64	2.1 ± 1.08	
(**b**)				

**Table 9:** Illustrates the results of the Wilcoxon signed-rank test analysis of VEPs under the effect of the six monochromatic filters compared to the baseline value. The Z and *p*-value are presented to show the strength of their impact on the neural activity of our participants.

N = 12	Alpha		Mu		Omega		Neurasthenic		Stimulant		Depressant	
	Z	*p*	Z	*p*	Z	*p*	Z	*p*	Z	*p*	Z	*p*
N75 of left eye					−2.90	0.004	−2.04	0.041				
P100 of left eye	−2.16	0.031			−2.59	0.010	−2.98	0.003				
VEP amplitude of left eye			−3.06	0.002	−3.06	0.002	−3.06	0.002	−2.67	0.008		
N75 of right eye	−3.06	0.002	−2.63	0.009	−2.98	0.003	−2.51	0.012				
P100 of right eye			−2.12	0.034			−2.82	0.005			−2.39	0.017
VEP amplitude of right eye			−2.20	0.028	−2.20	0.028	−2.27	0.023	−2.12	0.034		

Z (Z-value); *p* (*p*-value).

## Data Availability

The data presented in this study are available on request from the corresponding author. The data are not publicly available due to confidentiality.

## Abbreviations

The following abbreviations are used in this manuscript:

VEPs	visual evoked potentials
SA	strabismus and amblyopia
LAT	latency expressed in milliseconds (ms)
B–F	difference between the baseline value and the one obtained using the specific filter converted to percentage (%), where the (+) sign signifies an increment in the recorded value and the (−), decrease of the value
PP AMP	the amplitude measured from peak N75 to P100 expressed in microvolts (μV)
LAT DIFF LT-RT	difference between the P100 latency of the left and right eye expressed in milliseconds (ms)

## Appendix A

Clinical evaluation

Testing procedures were identical for all participants:

Motor clinical testing: The Cover–Uncover test at distance and near was performed to establish the direction and magnitude of strabismus, using the Spielmann translucent occluder, while the alternating cover test was performed to determine the phoria state of the non-strabismic participants. The deviation and the amount of phorias was neutralized with the Berens prism bar.

Sensorial clinical testing: Distant and near VA was measured using logMAR charts at 3 m and 40 cm, respectively to determine the presence or absence of amblyopias among participants. The Worth dot test was used to evaluate flat and peripheral fusion and to detect any suppression. It was performed using red-green lenses over the optical correction of the patient at two different distances: near and far.

The Random Dot test-2 was used to evaluate depth perception using contour (local) and global stimuli to measure stereopsis. The test was applied only at close distances with polarized glasses over the optical correction of the patient. The test detects disparities ranging from gross to fine stereopsis (500–12.5 s of arc).

The Monocular estimated method retinoscopy at 40 cm (MEM) was used as a complementary test to determine the best optical prescription during the subjective refraction of participants. Considering that the accommodation can affect the binocular system, the best way to prescribe is by introducing this variable into the equation.

## References

[1] Truong J.Q., Ciuffreda K.J., Han M.H.E., Suchoff I.B. (2014). Photosensitivity in mild traumatic brain injury (mTBI): A retrospective analysis. Brain Inj..

[2] Riddell P.M., Wilkins A., Hainline L. (2006). The Effect of Colored Lenses on the Visual Evoked Response in Children With Visual Stress. Optom. Vis. Sci..

[3] Friederichs E., Wahl S. (2017). (Re)-wiring a brain with light: Clinical and visual processing findings after application of specific coloured glasses in patients with symptoms of a visual processing disorder (CVPD): Challenge of a possible new perspective?. Med. Hypotheses.

[4] Huang J., Zong X., Wilkins A., Jenkins B., Bozoki A., Cao Y. (2011). fMRI evidence that precision ophthalmic tints reduce cortical hyperactivation in migraine. Cephalalgia.

[5] Hamm L.M., Black J., Dai S., Thompson B. (2014). Global processing in amblyopia: A review. Front. Psychol..

[6] Duan Y., Norcia A.M., Yeatman J.D., Mezer A. (2015). The Structural Properties of Major White Matter Tracts in Strabismic Amblyopia. Investig. Ophthalmol. Vis. Sci..

[7] Ouyang J., Yang L., Huang X., Zhong Y.L., Hu P.H., Zhang Y., Pei C.G., Shao Y. (2017). The atrophy of white and gray matter volume in patients with comitant strabismus: Evidence from a voxel-based morphometry study. Mol. Med. Rep..

[8] Shao Y., Li Q., Li B., Lin Q., Su T., Shi W., Zhu P., Yuan Q., Shu Y., He Y. (2019). Altered brain activity in patients with strabismus and amblyopia detected by analysis of regional homogeneity: A resting-state functional magnetic resonance imaging study. Mol. Med. Rep..

[9] Friedman D.S., Repka M.X., Katz J., Giordano L., Ibironke J., Hawse P., Tielsch J.M. (2009). Prevalence of Amblyopia and Strabismus in White and African American Children Aged 6 through 71 Months: The Baltimore Pediatric Eye Disease Study. Ophthalmology.

[10] Levi D.M., Knill D.C., Bavelier D. (2015). Stereopsis and amblyopia: A mini-review. Vis. Res..

[11] Niechwiej-Szwedo E., Chandrakumar M., Goltz H.C., Wong A.M.F. (2012). Effects of Strabismic Amblyopia and Strabismus without Amblyopia on Visuomotor Behavior, I: Saccadic Eye Movements. Investig. Ophthalmol. Vis. Sci..

[12] Sawamura H., Gillebert C.R., Todd J.T., Orban G.A. (2018). Binocular stereo acuity affects monocular three-dimensional shape perception in patients with strabismus. Br. J. Ophthalmol..

[13] Wallace L.B. (2009). The Theory and Practice of Syntonic Phototherapy: A Review. Optom. Vis. Dev..

[14] Hegedűs B., Viharos L., Gervain M., Gálfi M. (2009). The Effect of Low-Level Laser in Knee Osteoarthritis: A Double-Blind, Randomized, Placebo-Controlled Trial. Photomed. Laser Surg..

[15] Kothari R., Bokariya P., Singh S., Singh R. (2016). A Comprehensive Review on Methodologies Employed for Visual Evoked Potentials. Scientifica.

[16] Baiano C., Zeppieri M. (2023). Visual Evoked Potential. StatPearls.

[17] Aminoff M. (2004). Aminoff’s Electrodiagnosis in Clinical Neurology.

[18] Andrade E.P., Berezovsky A., Sacai P.Y., Pereira J.M., Rocha D.M., Salomão S.R. (2016). Dysfunction in the fellow eyes of strabismic and anisometropic amblyopic children assessed by visually evoked potentials. Arq. Bras. De Oftalmol..

[19] Halfeld Furtado de Mendonça R., Abbruzzese S., Bagolini B., Nofroni I., Ferreira E.L., Odom J.V. (2013). Visual evoked potential importance in the complex mechanism of amblyopia. Int. Ophthalmol..

[20] Hou C., Pettet M.W., Norcia A.M. (2008). Abnormalities of coherent motion processing in strabismic amblyopia: Visual-evoked potential measurements. J. Vis..

[21] Davis A.R., Sloper J.J., Neveu M.M., Hogg C.R., Morgan M.J., Holder G.E. (2008). Differential changes in color and motion-onset visual evoked potentials from both eyes in early- and late-onset strabismic amblyopia. Investig. Ophthalmol. Vis. Sci..

[22] Zheng X., Xu G., Zhi Y., Wang Y., Han C., Wang B., Zhang S., Zhang K., Liang R. (2019). Objective and quantitative assessment of interocular suppression in strabismic amblyopia based on steady-state motion visual evoked potentials. Vis. Res..

[23] Greenstein V.C., Eggers H.M., Hood D.C. (2008). Multifocal visual evoked potential and automated perimetry abnormalities in strabismic amblyopes. J. Am. Assoc. Pediatr. Ophthalmol. Strabismus.

[24] Heravian J., Daneshvar R., Dashti F., Azimi A., Ostadi Moghaddam H., Yekta A.A., Esmaily H. (2011). Simultaneous pattern visual evoked potential and pattern electroretinogram in strabismic and anisometropic amblyopia. Iran Red. Crescent Med. J..

[25] Willeford K.T., Fimreite V., Ciuffreda K.J. (2016). The effect of spectral filters on VEP and alpha-wave responses. J. Optom..

[26] Ibrahimi D., Mendiola-Santibañez J.D., Cruz-Martínez E., Gómez-Espinosa A., Torres-Pacheco I. (2021). Changes in the Brain Activity and Visual Performance of Patients with Strabismus and Amblyopia after a Compete Cycle of Light Therapy. Brain Sci..

[27] Ibrahimi D., Mendiola-Santibañez J.D., Martínez E.c., Rodríguez-Reséndiz J., Pacheco I.T. (2021). Cortical Activity at Baseline and During Light Stimulation in Patients With Strabismus and Amblyopia. IEEE Access.

[28] Argilés M., Sunyer-Grau B., Arteche-Fernandez S., Peña-Gómez C. (2022). Functional connectivity of brain networks with three monochromatic wavelengths: A pilot study using resting-state functional magnetic resonance imaging. Sci. Rep..

[29] Spitler H. (1941). The Syntonic Principle, Its Relation to Health and Ocular Problems.

[30] Scheiman M., Wick B., Steinman B. (2002). Clinical Management of Binocular Vision: Heterophoric, Accommodative, and Eye Movement Disorders.

[31] Commission Internationale de L’Eclairage (2011). 017/E: 2011 2011 ILV: International Lighting Vocabulary. https://www.amazon.com/CIE-017-International-lighting-vocabulary/dp/B008SVPK12.

[32] Duffau H. (2016). Brain Plasticity and Reorganization Before, During, and After Glioma Resection. Glioblastoma.

[33] Fan L., Li H., Zhuo J., Zhang Y., Wang J., Chen L., Yang Z., Chu C., Xie S., Laird A.R. (2016). The Human Brainnetome Atlas: A New Brain Atlas Based on Connectional Architecture. Cereb. Cortex.

[34] Gooley J., Saper C. (2017). Anatomy of the Mammalian Circadian System. Principles and Practice of Sleep Medicine.

[35] Gomes C.C., Preto S. (2015). Blue Light: A Blessing or a Curse?. Procedia Manuf..

[36] Salkind N. (2002). Child Development.

[37] Chéreau R., Williams L.E., Bawa T., Holtmaat A. (2022). Circuit mechanisms for cortical plasticity and learning. Semin. Cell Dev. Biol..

[38] Vandewalle G., Maquet P., Dijk D.J. (2009). Light as a modulator of cognitive brain function. Trends Cogn. Sci..

[39] Prayag A.S., Münch M., Aeschbach D., Chellappa S.L., Gronfier C. (2019). Light Modulation of Human Clocks, Wake, and Sleep. Clocks Sleep.

[40] Vandewalle G., Schmidt C., Albouy G., Sterpenich V., Darsaud A., Rauchs G., Berken P.Y., Balteau E., Degueldre C., Luxen A. (2007). Brain Responses to Violet, Blue, and Green Monochromatic Light Exposures in Humans: Prominent Role of Blue Light and the Brainstem. PLoS ONE.

